# Myocardium of patients with dilated cardiomyopathy presents altered expression of genes involved in thyroid hormone biosynthesis

**DOI:** 10.1371/journal.pone.0190987

**Published:** 2018-01-10

**Authors:** Carolina Gil-Cayuela, Ana Ortega, Estefanía Tarazón, Luis Martínez-Dolz, Juan Cinca, José Ramón González-Juanatey, Francisca Lago, Esther Roselló-Lletí, Miguel Rivera, Manuel Portolés

**Affiliations:** 1 Cardiocirculatory Unit, Health Research Institute of La Fe University Hospital (IIS La Fe), Valencia, Spain; 2 Members of the Center for Biomedical Research Network in Cardiovascular Diseases (CIBERCV), Madrid, Spain; 3 Heart Failure and Transplantation Unit, Cardiology Department, La Fe University Hospital, Valencia, Spain; 4 Cardiology Service of Santa Creu i Sant Pau Hospital, Barcelona, Spain; 5 Cellular and Molecular Cardiology Research Unit, Department of Cardiology and Institute of Biomedical Research, University Clinical Hospital, Santiago de Compostela, Spain; Scuola Superiore Sant'Anna, ITALY

## Abstract

**Background:**

The association between dilated cardiomyopathy (DCM) and low thyroid hormone (TH) levels has been previously described. In these patients abnormal thyroid function is significantly related to impaired left ventricular (LV) function and increased risk of death. Although TH was originally thought to be produced exclusively by the thyroid gland, we recently reported TH biosynthesis in the human ischemic heart.

**Objectives:**

Based on these findings, we evaluated whether the genes required for TH production are also altered in patients with DCM.

**Methods:**

Twenty-three LV tissue samples were obtained from patients with DCM (n = 13) undergoing heart transplantation and control donors (n = 10), and used for RNA sequencing analysis. The number of LV DCM samples was increased to 23 to determine total T4 and T3 tissue levels by ELISA.

**Results:**

We found that all components of TH biosynthesis are expressed in human dilated heart tissue. Expression of genes encoding thyroperoxidase (–2.57-fold, *P* < 0.05) and dual oxidase 2 (2.64-fold, *P* < 0.01), the main enzymatic system of TH production, was significantly altered in patients with DCM and significantly associated with LV remodeling parameters. Thyroxine (T4) cardiac tissue levels were significantly increased (*P* < 0.01), whilst triiodothyronine (T3) levels were significantly diminished (*P* < 0.05) in the patients.

**Conclusions:**

Expression of TH biosynthesis machinery in the heart and total tissue levels of T4 and T3, are altered in patients with DCM. Given the relevance of TH in cardiac pathology, our results provide a basis for new gene-based therapeutic strategies for treating DCM.

## Introduction

Dilated cardiomyopathy (DCM) is characterized by cardiac chambers dilation and ventricular dysfunction that frequently results in heart failure (HF), a condition that is associated with a high mortality rate [1]. The association between DCM and low thyroid hormone (TH) levels has been described [2–4]. Hypothyroidism is associated with decreased cardiac output due to impaired systolic and diastolic function [5,6]. Important changes in cardiac structure, and an increased risk of myocardial fibrosis have also been reported [7–9]. Consistent with this, TH replacement therapy has been shown to improve cardiovascular performance and ventricular remodeling in experimental models of hypothyroidism [10–12].

TH biosynthesis depends on complex machinery located in thyrocytes. Iodide is taken up through the Na/I symporter (NIS) and transported via pendrin from the cytoplasm into the follicular lumen, where iodide organification and coupling is catalyzed by dual oxidase (DUOX) and thyroperoxidase (TPO). H_2_O_2_ produced by DUOX is used by TPO to oxidize iodide and iodinate tyrosyl residues of thyroglobulin (TG), forming iodinated tyrosyl intermediates. TPO couples these intermediates to form the hormones T3 and T4, which are then endocytosed, hydrolyzed, and secreted into the blood [13,14]. The active form of TH is T3 and its availability is controlled by deiodinases, which catalyze its conversion from T4 [15].

Reduced TH levels may be a consequence of at least one of the following mechanisms: (a) alterations in TH receptors and/or transporters [16,17], (b) reduced TH production [18,19] or, (c) increased TH degradation [20,21].

We recently showed that all of the genes involved in TH biosynthesis are expressed in the human heart, and that their expression is altered in patients with ischemic cardiomyopathy [22]. Given these new data, the objective of the present study was to use RNA-sequencing (RNA-seq) technology to determine whether the genes required for TH biosynthesis are expressed in human dilated cardiomyopathy. We also sought to evaluate whether the expression levels of TH biosynthesis genes are altered in patients with DCM. Finally, we wanted to determine whether changes in TH expression are related to left ventricular (LV) remodeling parameters.

## Methods

### Sample collection

LV tissue samples for RNA-seq analysis were obtained from 23 explanted human hearts: 13 from patients with DCM, and 10 from non-diseased controls (CNTs). We increased the number of DCM heart samples to 23 in order to analyze total tissue levels of T3 (tT3) and T4 (tT4). We were permitted access to operating rooms during interventions, and explanted hearts were available in all cases, which reduced the time between reception and storage of samples. This ensured high-quality samples, as evidenced by RNA integrity numbers (RIN; ≥ 9). Tissue samples of approximately 5 cm^2^ were obtained from near the apex of the left ventricle of each explanted heart. Samples were maintained in 0.9% NaCl, and kept at 4°C for a maximum of 6 h after the loss of coronary circulation. Samples were stored at –80°C until use.

Clinical history, electrocardiograms, Doppler echocardiography and hemodynamic study results, and coronary angiography data were available for the patients. Non-ischemic DCM was diagnosed when patients showed intact coronary arteries by coronary angiography, and LV systolic dysfunction (ejection fraction; EF < 40%) with a dilated left ventricle (LV end-diastolic diameter; LVEDD > 55 mm). Patients with primary valvular disease were excluded from the study. Patients were classified according to the New York Heart Association functional criteria and were receiving medical treatment according to the guidelines of the European Society of Cardiology [23].

All controls showed normal LV function (EF > 50%), as determined by Doppler echocardiography, and had no history of cardiac disease. CNT samples were obtained from non-diseased donor hearts that had been rejected for cardiac transplantation because of size or blood type incompatibility, and the impossibility of finding a new recipient during the transplantation window. For these donors, the cause of death was either cerebrovascular events or motor vehicle accidents.

This investigation was approved by the Ethics Committee (Biomedical Investigation Ethics Committee of La Fe University Hospital of Valencia, Spain). Signed informed consent was obtained from each patient prior to tissue collection. The study was conducted in accordance with the guidelines of the Declaration of Helsinki [24].

### Echo-Doppler study

Echo-Doppler testing was performed at La Fe University Hospital, using standard echocardiographic systems equipped with 2.5- to 4-MHz transducers. Echocardiographic examinations were performed as described by Cortés et al [25].

### RNA extraction

Heart samples were homogenized with TRIzol® Reagent in a TissueLyser LT (Qiagen; UK). All RNA extractions were performed using a PureLink® RNA Mini Kit (Ambion Life Technologies; CA, USA), according to the manufacturer’s instructions. RNA was quantified using a NanoDrop1000 spectrophotometer (Thermo Fisher Scientific; UK) and the purity and integrity of RNA samples were measured using an Agilent 2100 Bioanalyzer with the RNA 6000 Nano LabChip kit (Agilent Technologies; Spain). As an established condition for inclusion in the study, all selected samples displayed a 260/280 nm absorbance ratio greater than 2.0 and RIN ≥ 9.

### RNA sequencing and computational analysis

RNA-seq procedures and analyses have been extensively described by Ortega et al.[26]. Gene expression levels were calculated using the HTSeq software (http://www-huber.embl.de/users/anders/HTSeq/; version 0.5.4p3). Poor quality reads (Phred score < 10) were eliminated using Picard Tool (http://picard.sourceforge.net/; version 1.83) and only unique reads were considered for gene expression level estimation. The edgeR method (version 3.2.4), was applied for analysis of differential expression between conditions [27]. This software allows normalization of RNA-seq data based on sequencing depth, GC content, and gene length; for analysis of differential expression, a Poisson model was used to estimate the variance of the RNA-seq data for different conditions. Resulting data units are usually referred to as arbitrary units (AU). The data described in the current study have been deposited in the NCBI Gene Expression Omnibus (GEO) and can be retrieved using the GEO Series accession number GSE55296 (http://www.ncbi.nlm.nih.gov/geo/query/acc.cgi?acc=GSE55296).

### RT-qPCR analysis

Reverse transcription was carried out using 1 μg of total RNA from 20 DCM and 10 CNT LV tissue samples and the M-MLV enzyme (Invitrogen, UK) according to the manufacturer’s protocol. RT-qPCR was performed in duplicate in a ViiA7 Fast Real-Time PCR System according to the manufacturer’s instructions (Applied Biosystems; USA). The following TaqMan probes were obtained from Thermo Fisher Scientific: *TPO* (Hs00892519_m1), *DUOX2* (Hs00204187_m1), and the housekeeping genes *GAPDH* (Hs99999905_m1), *PGK1* (Hs99999906_m1), and *TFRC* (Hs00951083_m1) were used as endogenous controls. Relative gene expression levels were calculated using the 2^−ΔΔCT^ method [28].

### Determination of tT4 and tT3 tissue levels

Thirty milligrams of frozen LV samples was transferred to Lysing Matrix D tubes designed for use with a FastPrep-24 homogenizer (MP Biomedicals, Santa Ana, USA). The tubes contained a total protein extraction buffer (2% SDS, 10 mM EDTA, 6 mM Tris-HCl, pH 7.4) with protease inhibitors (25 μg/mL aprotinin and 10 μg/mL leupeptin). Homogenates were centrifuged at 15,000 ×*g* for 10 min at 4°C, and supernatants were aliquoted.

tT4 and tT3 levels were measured using a competitive enzyme-linked immunosorbent assay (ELISA) kit (tT4: ab178664; tT3: ab108685) (Abcam, Cambridge, UK). For each assay, we used 30 μL of supernatant. Sensitivity limits for the tT4 and tT3 assays were 0.004 μg/mL and 0.05 ng/mL respectively. Cross-reactivity with triiodothyronine (100 μg/mL) in the tT4 assay was < 0.03, and with thyroxine (10 μg/dL) in the tT3 assay was < 0.01. Reproducibility of the tT4 assay had a CV inter-assay of 6.0% ± 2.2% and intra-assay of 4.8% ± 1.6%. Reproducibility of tT3 had a CV inter-assay of 4.9% ± 2.3% and intra-assay of 3.9% ± 1.6%. Recovery of 25–50 μg/mL tT4 added to cardiac homogenates gave an average value of 96.5% ± 4.5% with reference to original concentrations. Recovery of 1–2 ng/mL tT3 added to cardiac homogenates gave an average value of 96.9% ± 5.2% with reference to original concentrations. Absorbance was measured on a Tecan Sunrise microplate reader (Männedorf, Switzerland) at 450 nm with a reference wavelength of 620 nm, and data were quantified using Magellan software (version 6.4; Tecan).

### Statistical analysis

Data are presented as mean ± standard deviation for continuous variables and as a percentage for discrete variables. The Kolmogorov-Smirnov test was used to test variables for normal distribution. Between-group comparisons of clinical characteristics were computed using the Student’s *t*-test (for continuous variables) or Fisher’s exact test (for discrete variables). Between-group comparisons of tissue mRNA and hormone levels were performed using the Student’s *t*-test (for variables with a normal distribution) or the Mann-Whitney U test (for variables with a non-normal distribution). Pearson’s correlation coefficient was used to examine associations between clinical parameters and mRNA and hormone levels (normally distributed variables); Spearman’s correlation coefficient was computed for non-normally-distributed variables. A *P*-value of < 0.05 was considered statistically significant. Genes with a fold change > 1.5 were considered differentially expressed. All statistical analyses were performed using SPSS software v. 20 for Windows (IBM SPSS Inc., Chicago, IL, USA).

## Results

### Clinical characteristics

DCM patients were mostly men for both RNA-seq (92%) and hormone (70%) analyses, with a mean age of 51 ± 11 years and 51 ± 13 years, respectively. The patients belonged to classes III–IV according to the New York Heart Association functional classification and had been previously diagnosed with significant comorbidities. The CNT group also consisted mainly of men (80%), with a similar mean age (47 ± 16 years). Comorbidities and other echocardiographic data were not available for the CNT group, in accordance with the Spanish Organic Law on Data Protection 15/1999. Clinical characteristics of the patients are summarized in Table 1.

**Table 1:** Clinical characteristics of patients with dilated cardiomyopathy.

	RNA-seq	TH levels	
	DCM (n = 13)	DCM (n = 23)	*P*-value
Age (years)	51±11	51±13	0.992
Gender male (%)	92	70	0.115
NYHA class	3.4±0.4	3.3±0.4	0.653
BMI (kg/m^2^)	27±5	26±5	0.378
Total cholesterol (mg/dL)	147±37	133±41	0.393
Prior hypertension (%)	17	17	1.0
Prior diabetes mellitus (%)	17	20	0.815
Hemoglobin (mg/mL)	13±3	12±3	0.474
Hematocrit (%)	39±8	37±9	0.445
Duration of disease (months)	75±68	77±66	0.933
***Echo-Doppler study***			
EF (%)	20±7	21±10	0.578
LVESD (mm)	71±12	68±12	0.265
LVEDD (mm)	80±11	76±11	0.216
LVMI (g/m^2^)	241±77	216±68	0.245

Data are showed as the mean value ± standard deviation. DCM, dilated cardiomyopathy; TH, thyroid hormone; NYHA, New York Heart Association; BMI, body mass index; EF, ejection fraction; LVESD, left ventricular end-systolic diameter; LVEDD, left ventricular end-diastolic diameter; LVMI, left ventricular mass index.

### Gene expression analysis

**Fig 1:**
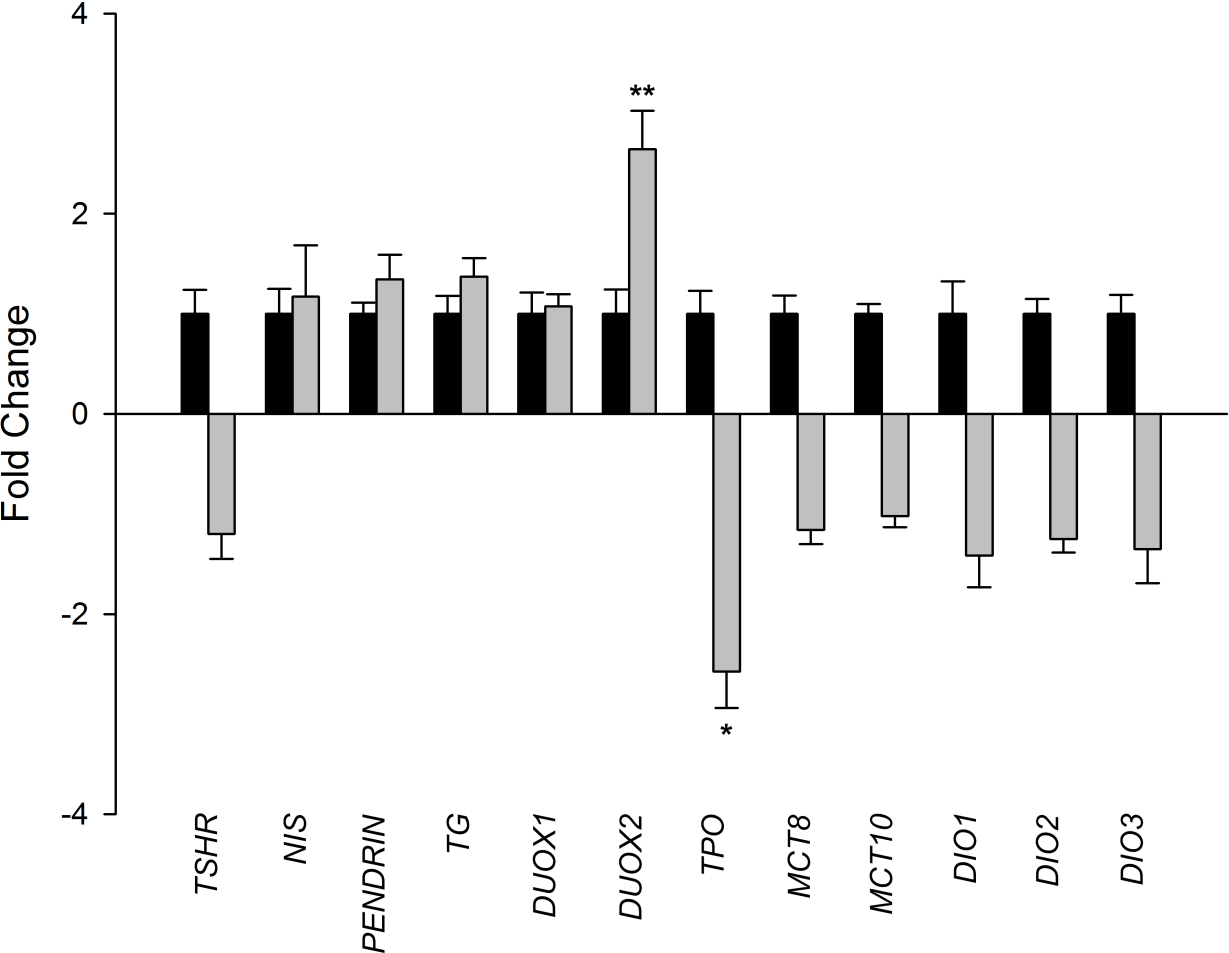
Expression levels of thyroid hormone biosynthesis-related genes in dilated hearts. Comparison of the mRNA levels of thyroid hormone biosynthesis-related genes in dilated hearts (n = 13; grey bar) vs. controls (n = 10). Control values were set to 1. Results are presented as fold change ± standard error of the mean. *TSHR*: thyroid stimulating hormone receptor; *NIS*: Na/I symporter; *TG*: thyroglobulin; *DUOX1*: dual oxidase 1; *DUOX2*: dual oxidase 2; *TPO*: thyroperoxidase; *MCT8*: monocarboxylate transporter 8; *MCT10*: monocarboxylate transporter 10; *DIO1*: deiodinase 1; *DIO2*: deiodinase 2; *DIO3*: deiodinase 3. Results were considered statistically significant at *P < 0.05 and **P < 0.01.

In order to investigate transcriptome-level differences between CNT (n = 10) and DCM (n = 13) samples, a large-scale screening study was performed using RNA-seq technology. Comparison of CNT and DCM samples revealed 1628 differentially expressed genes (596 upregulated, ≥1.5-fold and 1032 downregulated, ≥1.5-fold decrease, *P* < 0.05 for all). We focused on TH biosynthesis-related genes, and detected expression of all the components required for TH production (Table 2), as established by Meischl et al [29]. These, have not previously been reported in human DCM. In particular, comparison of DCM and CNT samples showed that *TPO* (–2.57-fold; *P* < 0.05) and *DUOX2* (2.64-fold; *P* < 0.01) were differentially expressed. Representative fold-changes are shown in Fig 1.

**Table 2:** Expressed thyroid hormone biosynthesis-related genes in patients with dilated cardiomyopathy.

*Gene*	CNT (n = 10)	DCM (n = 13)
	Mean ± SD	Mean ± SD
***TSHR***	2.37 ± 1.26	1.98 ± 1.24
***SLC5A5***	1.57 ± 0.78	1.83 ± 1.80
***SLC26A4***	9.57 ± 3.40	12.87 ± 8.45
***TG***	4.01 ± 2.02	5.50 ± 2.66
***DUOX1***	11.00 ± 7.04	11.81 ± 4.58
***DUOX2***	5.63 ± 3.87	14.87 ± 7.78**
***TPO***	96.51 ± 65.94	37.49 ± 18.43*
***SLC16A2***	67.35 ± 38.93	58.18 ± 26.07
***SLC16A10***	29.09 ± 8.99	28.48 ± 11.41
***DIO1***	1.83 ± 1.18	1.29 ± 0.76
***DIO2***	195.94 ± 90.53	156.77 ± 59.83
***DIO3***	1.77 ± 0.74	1.31 ± 0.87

Data are showed as the mean value (arbitrary units) ± standard deviation (SD). *TSHR*: thyroid stimulating hormone receptor; *SLC5A5*: Na/I symporter (NIS); *SLC26A4*: pendrin; *TG*: thyroglobulin; *DUOX1*: dual oxidase 1; *DUOX2*: dual oxidase 2; *TPO*: thyroperoxidase; *SLC16A2*: monocarboxylate transporter 8 (MCT8); *SLC16A10*: monocarboxylate transporter 10 (MCT10); *DIO1*: deiodinase 1; *DIO2*: deiodinase 2; *DIO3*: deiodinase 3.

*P < 0.05

**P < 0.01.

### RT-qPCR analyses

To confirm changes in the expression of both *TPO* and *DUOX2* genes between DCM and CNT groups, we performed RT-qPCR analyses. In accordance with the previously measured mRNA levels, RT-qPCR results showed significant *TPO* downregulation (–1.50-fold; *P* < 0.05) and *DUOX2* upregulation (2.02-fold; *P* < 0.05).

### tT4 and tT3 tissue levels

**Fig 2:**
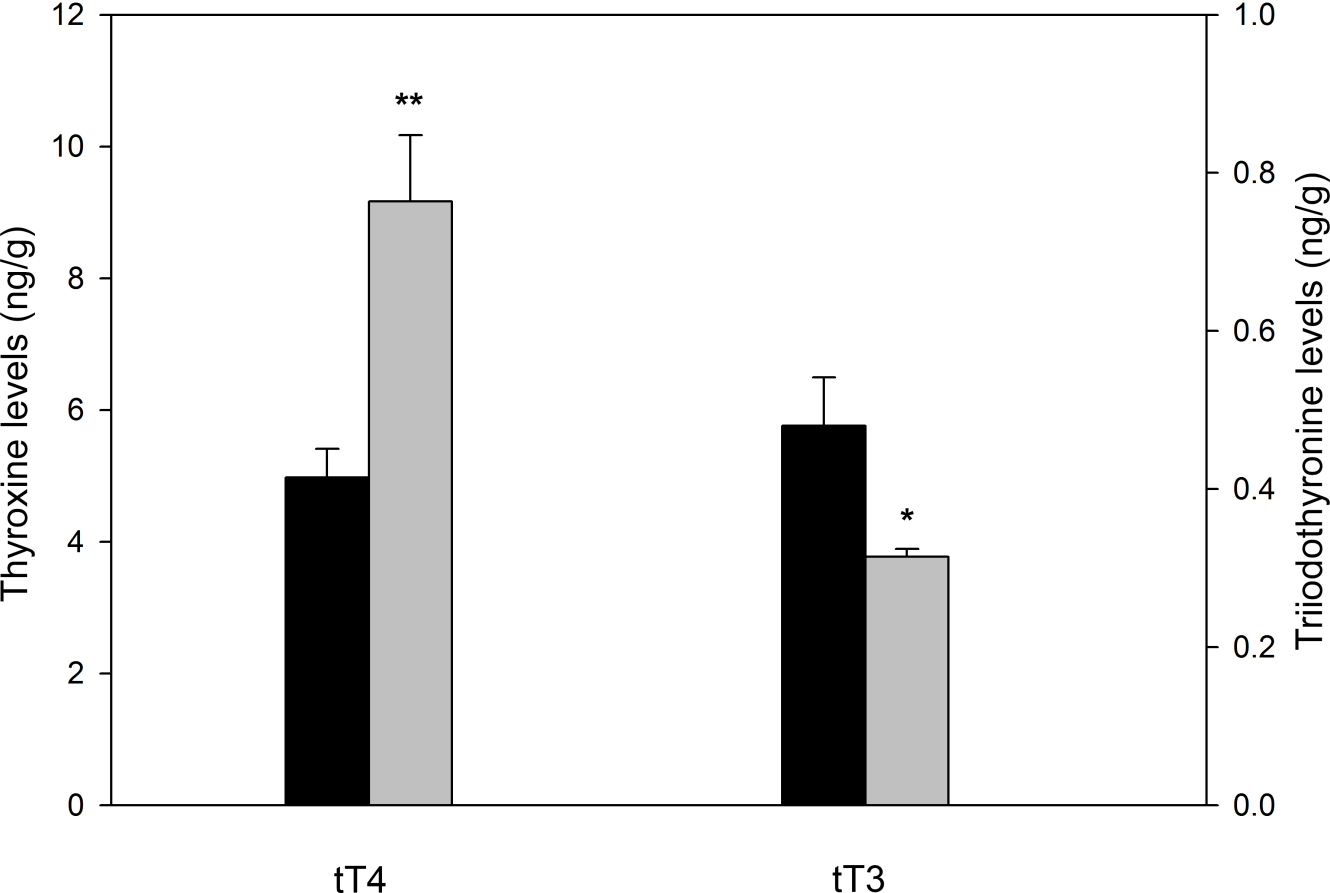
Total tissue levels of T4 and T3 in patients with dilated cardiomyopathy. Comparison of total cardiac thyroxine (tT4) and triiodothyronine (tT3) in dilated hearts (n = 23; grey bar) vs. controls (n = 10). Results are presented as mean concentration (ng/g) ± standard error of the mean. Results were considered statistically significant at *P < 0.05 and **P < 0.01 vs. CNT.

We performed competitive ELISAs to determine tT4 and tT3 cardiac tissue levels. tT4 was increased in the DCM group compared to the CNT group (9.17 ± 4.02 vs. 4.98 ± 1.37 ng/g, *P* < 0.01). In contrast, tT3 levels were significantly decreased in the DCM group compared to the CNT group (0.31 ± 0.01 vs. 0.48 ± 0.06 ng/g, *P* < 0.05) (Fig 2).

### Relationships between TH expression levels and LV remodeling parameters

To investigate the relationship between the expression of specific genes and remodeling, we searched for correlations between *TPO* and *DUOX2* mRNA levels and LV remodeling parameters. Interestingly, we observed significant positive correlations between *DUOX2* gene expression and LV end-systolic diameter (LVESD), LVEDD, and LV mass index (r = 0.643, *P* < 0.05; r = 0.739, *P* < 0.01; and r = 0.594, *P* < 0.05, respectively). However, no significant correlation was found between LV remodeling parameters and *TPO* gene expression.

## Discussion

TH is a critical regulator of cardiac growth and development, and its homeostasis is essential for optimal function of the cardiovascular system [30,31]. A primary change in HF is known as low-T3 syndrome, and is characterized by a reduction in tT3 levels. This condition occurs in approximately one-third of patients with advanced HF and is a strong predictor of mortality [32–34].

Although TH was traditionally thought to be exclusively produced by the thyroid gland, several studies have reported TH synthesis in thyroidectomized rats, cardiomyoblasts, and recently in the heart tissue from patients with ICM [22,29,35]. Cardiac TH biosynthesis may allow initiation of TH-protective mechanisms before local blood flow is restored. TH can limit apoptosis and stimulate angiogenesis after myocardial infarction by balancing pro-apoptotic and pro-survival signals [36]. Furthermore, TH improves cardiac contractility by increasing inhibition of phospholamban and allowing disinhibition of SERCa^2+^ activity [30,37]. It also prevents fibrosis and induces LV ellipsoidal reshaping by regulating collagen and MMP expression, among others mechanisms [9,38,39].

It has been reported that the main enzymatic system of the TH biosynthesis machinery (TPO and DUOX2) is altered in ICM, and that tT3 hormone levels are also decreased. In addition, differentially *TPO* expression is significantly related to pathological remodeling parameters and presents an altered methylation pattern that may influence the synthesis of the enzyme [22]. Thyroid iodide organification and coupling depends on both TPO and DUOX activity. DUOX2 produces H_2_O_2_, which is used by TPO to oxidize iodide. Iodine is then used to iodinate thyroglobulin (TG), forming tyrosyl intermediates that couple to originate T4 and T3 hormones [13,14]. Thus, alterations in TG iodination may be responsible for changes in tissue TH levels.

Based on all these previous findings, we sought to determine whether the genes required for TH biosynthesis are expressed in heart tissue from patients with DCM, and whether their expression is altered and related to ventricular dysfunction. Using RNA-seq, we measured the expression of all components required for TH biosynthesis (Fig 1). Consistent with previous data from patients with ICM, we found significantly altered and opposing expression of *TPO* and *DUOX2*. We also observed that changes in *DUOX2* expression were related to ventricular remodeling. Increased *DUOX2* mRNA levels and decreased *TPO* mRNA levels could result in high levels of H_2_O_2_ that would cause cardiomyocyte damage and apoptosis [40]. Therefore, these results suggest that reduced TH levels may be a consequence of changes in TH biosynthesis. In addition, we observed an increase in tT4 cardiac tissue levels, while tT3 levels were decreased. This is likely to reflect an additional problem in the deiodination process. We found that *DIO1*, *DIO2*, and *DIO3* levels were diminished by 20 to 30%, but these differences were not significant. Alterations in deiodinases have, however, been described in animal models of heart disease [41,42].

Local cardiac production of TH may be a protective mechanism of cardiac function in patients with DCM. Changes in the cardiac biosynthesis of TH may be the result of alteration in both the biosynthesis machinery and in the deiodination process, preventing the protective actions of TH. Restoring these mechanisms may lead to a recovery of T3 production and, therefore, to ameliorated LV function as a consequence of activation of the protective signaling pathways that limit damage, and thus improve cardiac contractility and morphology. The development of therapies that allow the reestablishment of TH cardiac production could be a good starting point for new medical strategies to treat patients with DCM.

### Study limitations

Patients who participated in this study were undergoing pharmacological treatments, some of which could have influenced the results. *TPO* and *DUOX2* expression has been validated by RT-qPCR, nevertheless protein or enzyme activity of these genes has not been measured. Because tissue samples were collected over a long period of time, LV remodeling parameters were not assessed using a state-of-the-art method; instead, we used 2D Doppler echocardiography.

## Conclusions

In the present study, we showed that all genes involved in TH biosynthesis are expressed in the myocardium of patients with DCM. mRNA levels of the main enzymatic system of this biosynthesis machinery were altered, and in particular, differential expression of *DUOX2* was significantly related to pathological remodeling parameters. Tissue levels of tT4 and tT3 were both significantly altered in patients with DCM. Therapeutic targeting of the cardiac TH biosynthesis machinery may provide protection against H_2_O_2_–induced damage, and in turn would reestablish TH production and improve LV performance.
